# Torque Measurement and Control for Electric-Assisted Bike Considering Different External Load Conditions

**DOI:** 10.3390/s23104657

**Published:** 2023-05-11

**Authors:** Ping-Jui Ho, Chen-Pei Yi, Yi-Jen Lin, Wei-Der Chung, Po-Huan Chou, Shih-Chin Yang

**Affiliations:** 1 Department of Mechanical Engineering, National Taiwan University, Taipei 106319, Taiwan; d09522012@ntu.edu.tw (P.-J.H.); f09522808@ntu.edu.tw (C.-P.Y.); f08522816@ntu.edu.tw (Y.-J.L.); 2 Industrial Technology Research Institute (ITRI), Hsinchu 310401, Taiwan; weider@itri.org.tw (W.-D.C.); phchou@itri.org.tw (P.-H.C.)

**Keywords:** electric-assisted bicycle, permanent magnet motor, two-wheeler simulation, E-bike pedaling power, E-bike cycling quality

## Abstract

This paper proposes a novel torque measurement and control technique for cycling-assisted electric bikes (E-bikes) considering various external load conditions. For assisted E-bikes, the electromagnetic torque from the permanent magnet (PM) motor can be controlled to reduce the pedaling torque generated by the human rider. However, the overall cycling torque is affected by external loads, including the cyclist’s weight, wind resistance, rolling resistance, and the road slope. With knowledge of these external loads, the motor torque can be adaptively controlled for these riding conditions. In this paper, key E-bike riding parameters are analyzed to find a suitable assisted motor torque. Four different motor torque control methods are proposed to improve the E-bike’s dynamic response with minimal variation in acceleration. It is concluded that the wheel acceleration is important to determine the E-bike’s synergetic torque performance. A comprehensive E-bike simulation environment is developed with MATLAB/Simulink to evaluate these adaptive torque control methods. In this paper, an integrated E-bike sensor hardware system is built to verify the proposed adaptive torque control.

## 1. Introduction

E-bikes have become popular for commuters due to the progress in AC motors and battery modules. By adding high-torque-density PM motors and lithium-ion batteries, E-bikes can provide more cycling power under the same weight. In general, electric bikes are categorized into two different systems. These are throttle-manipulated E-bikes and cycling-assisted E-bikes [1]. Considering throttle E-bikes, the motor torque output is controlled by the throttle on the handlebar. Because the throttle is directly manipulated by cyclists, safety issues can occur once the motor torque is sufficiently high. By contrast, cycling-assisted E-bikes automatically provide the motor torque, and the output value is dependent on the human pedaling torque. Compared to throttle E-bikes, cycling-assisted E-bikes have the advantage of safe riding behavior. By properly designing the motor torque, the human pedaling torque can be greatly reduced, especially under climbing and acceleration conditions.

It is not an easy task to determine suitable motor-assisted torques among all the different load conditions. In [2], a constant torque control strategy is proposed for E-bikes. Considering different road conditions, three to five different motor torque levels can be manually selected. In addition to being able to manually control the motor torque level, an electric bicycle can apply asymmetric assistance to the crank. The provision of motor torque at a specific crank angle has been proposed, which can aid patients with lower limb asymmetric function, such as post-stroke patients, so that the pedaling torque of the target leg is reduced [3]. However, this fixed torque control might not be suitable for the cyclist, considering pedaling torque variation.

In [4,5,6,7], an instantaneous pedaling torque waveform is analyzed. According to the circular motion theory, the cyclist’s pedaling torque should be a rectified sinusoidal waveform dependent on the bike pedal’s crank position. Under this effect, the motor torque can be designed as a rectified sinusoidal waveform similar to the pedaling torque. Compared to constant torque control, a smaller PM motor can be used to provide the same assisted performance. The pedaling torque might not fully contribute to the E-bike’s wheel torque. As reported in [8,9,10,11,12,13,14], an effective pedaling torque can be different depending on different crank positions.

It has been noted that cycling quality is different with respect to the different physiological factors of cyclists. The sources in [15,16,17,18,19,20,21] detail that cycling quality can be affected by various factors. These include the cyclist’s sex, purpose, cadence, speed, acceleration, vibration, experience, as well as the weather. Considering these factors, two platforms for riding performance have been derived. One is a performance index called the rating of perceived exertion (RPE), which was developed to command a suitable torque output [22], and the other makes additional use of the rider’s ability level, the E-bike’s characteristics (power, battery, weight), and the route profile (gradient and distance) to determine the output torque of the assist motor. What is unique is that the latter is built as a social platform. If the rider sets the motor’s output torque lower than the algorithm recommends, the rider will be able to earn more rewards [23].

Instead of a motor torque for pedaling torque reduction, a motor-assisted torque can also be implemented to achieve better physiological functions for the cyclist. In [24], the motor torque was manipulated with respect to the cyclist’s heart rate for a better physiological effect. However, this assisted method requires a high controller computation burden. In addition, because the E-bike frame weight is expected to be low, the cyclist’s weight and pedaling behavior might greatly affect the motor-assisted torque. In [25], the overall cycling mechanical powers were compared with two different cyclists of different weights: 95 kg and 50 kg. The resulting power consumption between the two cyclists differed by more than 50%. In [26], a comprehensive monitoring system was developed. This system integrates environmental factors [27,28], the cyclist’s heart rate [29,30] and respiratory rate [31], power consumption [32] and electromyogram [33,34] information, and journey time. Collecting data from the cloud can give the rider a reference indicator to determine the motor power of the journey in order to retain a longer battery life. The authors of [35] discuss the external load caused by the cyclist under climbing conditions. A suitable cycling performance was determined with the knowledge of various climbing-related load conditions. Instead of providing assisted torque, the recharge control can also be used to store the cyclist’s pedal power for better battery usage [36,37]. During low cyclist cadence, the stored mechanical power is returned for assisted torque to improve the cyclist’s blood oxygen and physiological stability. It is noteworthy that the feedback-based motor control can be implemented for assisted E-bike applications. The authors of [38] developed an improved feedback controller based on differential equations. In addition, a predictive feedback controller can be designed according to time-varying load conditions [39].

From a review of the existing references, key findings are summarized in Table 1. The sources in [1,2,24,38,39] aim to design suitable torque controllers for assisted E-bikes; however, no further analysis of the influence of the cyclist’s pedaling torque was addressed. By contrast, [4,5,6,7,8,9,10,11,12,13,14] investigate the cyclist’s pedaling dynamic with no motor-assisted torque assumed. Moreover, [15,16,17,18,19,20,21,22,23,25,26,27,28,29,30,31,32,33,34,35] focus on E-bike cycling performance with respect to human behaviors, including heartbeat, gender, and weight. The authors of [40,41] further evaluate recharge control for assisted motor output. Although several torque control methods have been proposed for E-bikes, a comprehensive analysis of different control methods is required with respect to various cycling load conditions.

To overcome these limitations on existing E-bike assisted torque control, this paper’s motivation is to find the best-suited assisted torque considering external loads. It is shown that the overall cycling torque is affected by external loads, including the cyclist’s weight, wind resistance, rolling resistance, and the road slope. Under these effects, the motor torque should be controlled with respect to these riding conditions. Four torque control methods are compared considering the dynamic effect on cycling torque and wheel acceleration. It is concluded that the wheel acceleration is important to determine the overall synergetic torque performance. The acceleration variation can be reduced by regulating the motor torque with the opposite phase as the human pedaling torque. All these torque control methods are evaluated with an E-bike simulation based on MATLAB/Simulink. An experimental bench is built to verify these methods.

## 2. E-Bike Pedaling Dynamic

This section discusses a dynamic model for an assisted E-bike. The pedaling behavior of cyclists is first discussed. After that, external disturbances’ torque loads are considered for the development of a dynamic E-bike model.

### 2.1. Cyclist Pedaling Behavior

**Figure 1:**
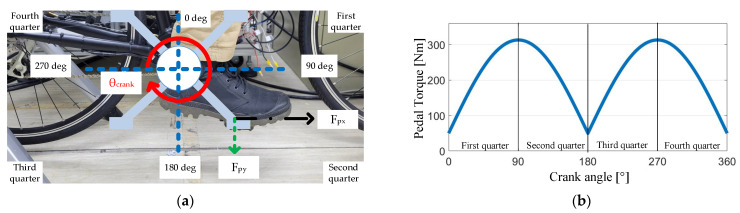
Relationship between crank position and pedaling torque: (**a**) pedaling torque component $F_{py}$/$F_{px}$ and crank position; (**b**) pedaling torque with respect to the crank position (no horizontal pedal force is assumed for simplicity).

Figure 1a shows cyclist pedaling behavior during bike riding. Ideally, the cyclist’s legs should be perpendicular to the horizontal ground. Under this effect, both of the cyclist’s feet are aligned with the pedal’s central axis. In this case, only the pedaling vertical force $F_{py}$ is generated. However, if the cyclist’s foot is not aligned with the pedal, the pedaling horizontal force $F_{px}$ might result in the degradation of the overall cycling performance, e.g., a reduction in pedaling torque and vibration.

### 2.2. Pedal Crank Angle Effort

Figure 1a also illustrates the relationship between the pedaling force $F_{py}$/$F_{px}$ and the crank angle position. In general, the cyclist’s pedaling force can be categorized into four different regions depending on the crank position. In Figure 1a, the bike is assumed to move forward to the right-hand side. In this case, the pedaling force in the first and second quarters are contributed by the cyclist’s right foot.

In Figure 1b, the first quarter is defined for a pedal position between 0–90°. Ideally, at a 0° crank position, the vertical force $F_{py}$ cannot contribute to the pedaling torque $T_{pdl}$. As a result, the overall $T_{pdl}$ should be zero at 0°, as seen in Figure 1b. By contrast, at 90°, a peak $T_{pdl}$ should appear, because $F_{py}$ is perpendicular to the crank. Similar cyclist pedaling behavior can be found in the second quarter.

Instead of the right foot reflecting the pedaling torque in the first and second quarters, the pedaling torque is generated by the left foot during the third and fourth quarters. Considering the third quarter for a crank position between 180–270° in Figure 1a, the corresponding pedaling torque $T_{pdl}$ is illustrated in Figure 1b. A similar pedaling torque $T_{pdl}$ can be analyzed in the fourth quarter. It is seen that the pedaling torque $T_{pdl}$ is equivalent to a sinusoidal waveform after the rectifier. The torque equation is formulated by:

(1) $$T_{pdl}=|F_{py}\times R_{crank}\times \sin\theta_{crank}|$$

where $R_{crank}$ and $\theta_{crank}$ are, respectively, the crank rotating radius and position. For the torque equation in (1), an ideal circular motion must be assumed, in which no horizontal force $F_{px}$ is generated by the cyclist. However, depending on different cyclist behaviors, $F_{px}$ might occur considering the external loads during cycling, resulting in a reduction in riding efficiency.

### 2.3. Parameters of Analyzed E-Bike

For assisted E-bikes, the total synergetic cycling torque consists of the cyclist’s pedaling torque and the motor-assisted torque. Table 2 lists the key parameters for the analyzed E-bike. In this table, the assisted motor is assumed to be installed in the rear wheel. This in-wheel motor can directly drive the wheel, avoiding torque loss due to the transmission and gear.

## 3. E-Bike Dynamics

**Figure 2:**
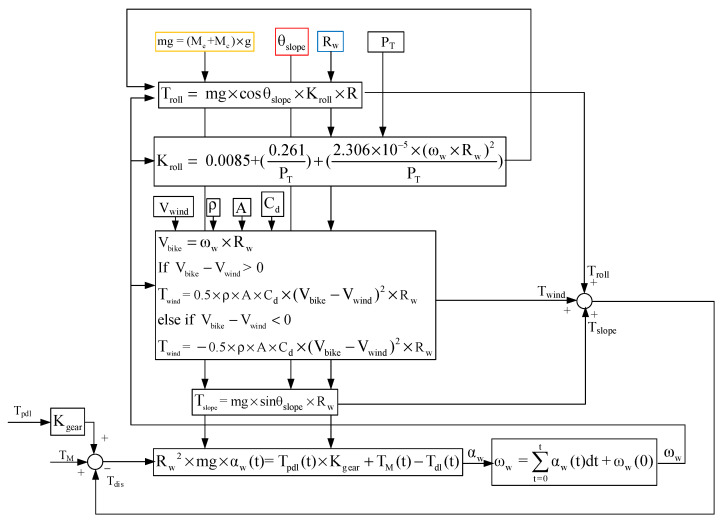
Block diagram of E-bike torque management signal process.

This section analyzes the wheel angular speed ω_w_ and acceleration α_w_ of the E-bike considering different torque control methods with external loads. An analytical E-bike model in Figure 2 is developed to investigate the ω_w_ and α_w_ performance of the E-bike under these external loads. These external loads include the wheel friction torque T_roll_, the windage torque T_wind_, and the climbing-reflected torque T_slope_. It can be shown that:

(2) $$T_{dis}=T_{roll}+T_{wind}+T_{slope}$$

where T_dis_ is the summation of all external loads. In addition, the road slope angle θ_slope_, the angular speed and acceleration ω_w_ and α_w_, the tire pressure P_T_, the bike wheel radius R, the wind speed V_wind_, the E-bike mass M_e_, the cyclist mass M_c_, the gravitational constant g, the density of air ρ, the aerodynamic drag coefficient $C_{d}$, and the frontal area A are all parameters used for the calculation of external loads. The maximum climbing angle of the E-bike can also be obtained under a specific value for T_pdl_ and T_M_.

### 3.1. Synergetic Torque

**Figure 3:**
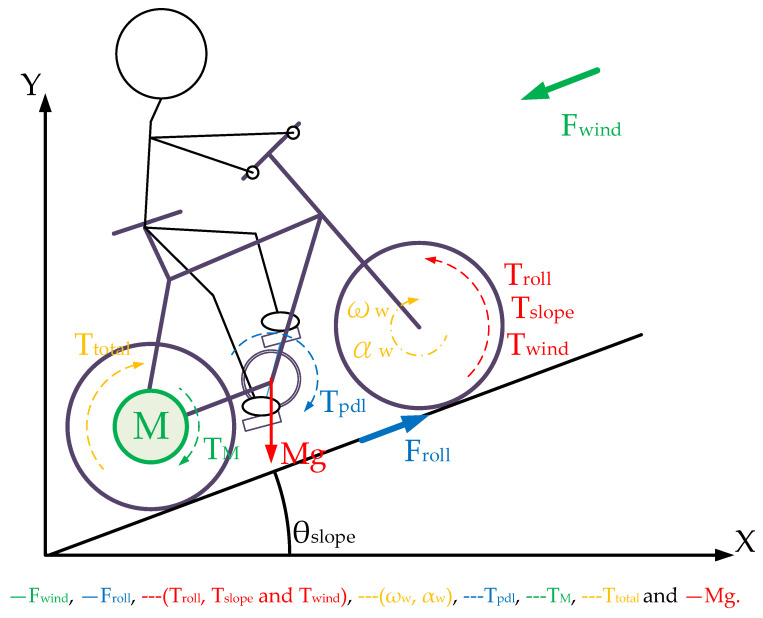
Free-body diagram of E-bike system with external loads.

In this paper, a two-degree-of-freedom E-bike model is realized, in which the E-bike is assumed to move forward or backward with different climbing angles. Figure 3 illustrates the corresponding E-bike free-body diagram considering these forces and torques in Figure 2. Considering the rigid body assumption in Figure 3, the synergetic torque T_total_ combines the cyclist pedaling torque T_pdl_ and the motor-assisted torque T_M_. It can be calculated by:

(3) $$T_{total}{=T}_{pdl}\times K_{gear}+T_{M}$$

T_total_ is assumed to drive the rear wheel. It is noted that the pedaling torque generated by the cyclist is assumed to be a rectified sinusoidal torque, illustrated in Figure 1b. Under this effect, the synergetic T_total_ can be either the constant torque or the sinusoidal torque, depending on the manipulation of the motor torque T_M_.

### 3.2. Wheel Friction Torque

This section discusses the wheel friction-reflected torque load. Considering the wheel friction, rolling without slipping is typically assumed for the wheel’s rotation. In general, the wheel friction might result in the friction-reflected torque T_roll_ on the overall cycling torque output. This is given by:

(4) $$T_{roll}=mg\times {\cos\theta }_{\mathrm{slope}}\times K_{\mathrm{roll}}\times R_{w}$$

where mg is the equivalent mass including the cyclist and E-bike, and θ_slope_ is the road slope angle in Figure 3. In addition, R is the E-bike rolling radius in Table 2. K_roll_ is the resistance coefficient affected by the road’s surface shape, the tire’s structure, material, and pressure, as well as the wheel speed.

In addition, the rolling resistance coefficient K_roll_ is strongly influenced by tire pressure. The tire deformation is visible with considerable rolling resistance when the tire pressure is low [41,42]. In general, the resistance coefficient is calculated by:

(5) $$K_{roll}=\;0.0085+(\frac{0.261}{P_{T}})+(\frac{2.306\times 10^{-5}\times {\left(\omega_{w}\times R_{w}\right)}^{2}}{P_{T}})$$

where ω_w_ is the wheel angular speed, and P_T_ is the tire pressure.

**Figure 4:**
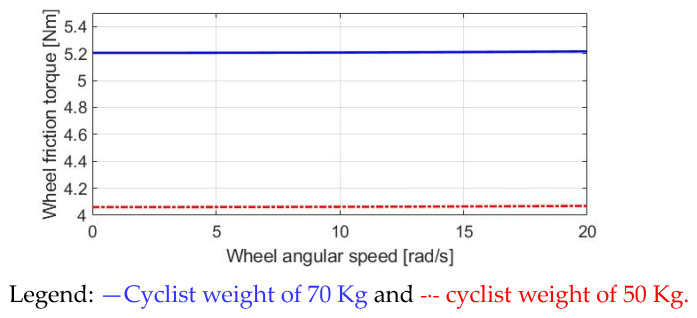
Analysis of wheel friction torque in E-bike.

Figure 4 illustrates T_roll_ versus the wheel speed. In this simulation, a constant acceleration of 1.334 rad/s^2^ is assumed, in which the wheel speed is increased from 0 to 20 rad/s within 15 s. Two cyclists, weighing 70 kg and 50 kg, are compared. Although the friction torque T_roll_ is slightly increased as the wheel speed increases, the influence of the cyclist’s weight is more visible than the wheel speed. Based on Figure 4, it can be concluded that T_roll_ is mainly dominated by the cyclist’s weight. Thus, the motor-assisted torque T_M_ can be manipulated depending on the current cyclist’s weight.

### 3.3. Windage Torque

This section explains the windage-reflected torque. Instead of the wheel friction torque, the airflow can cause aerodynamic resistance on both cyclists and E-bikes. On this basis, the airflow results in the windage torque T_wind_, which is shown to be:

(6) $$\begin{array}{l} V_{bike}=\omega_{w}\times R_{w}\\ \mathrm{If}\;V_{bike}-V_{wind}>\;0\\ T_{wind}=0.5\times \rho \times A\times C_{d}\times {\left(V_{bike}-V_{wind}\right)}^{2}\times R_{w}\\ \mathrm{else}\;\mathrm{if}\;V_{bike}-V_{wind}<0\\ T_{wind}=-0.5\times \rho \times A\times C_{d}\times {\left(V_{bike}-V_{wind}\right)}^{2}\times R_{w} \end{array}$$

where V_bike_ is calculated by the wheel angular speed, and V_wind_ is the corresponding wind speed depending on the airflow condition. In addition, ρ is the air density, $C_{d}$ is the aerodynamic drag coefficient, and A is the frontal area of airflow. For the analyzed E-bike in this paper, these three parameters are listed in Table 2.

**Figure 5:**
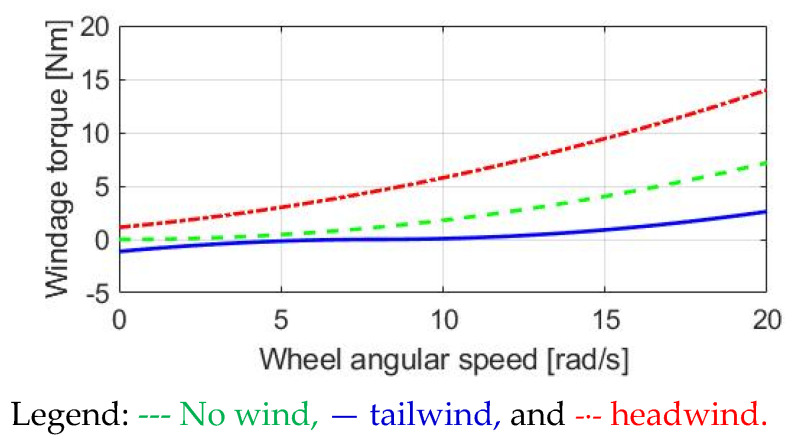
Analysis of windage torque in E-bike.

Figure 5 depicts the windage torque as the E-bike’s speed increases. In this calculation, a constant 1.334 rad/s^2^ acceleration is assumed. Within 15 s, the wheel angular speed is increased from 0 to 20 rad/s. In the case of no wind, the windage torque is equivalent to a quadratic function proportional to $v_{bike}^{2}$. Even at zero wheel speed V_bike_ = 0, there is a windage T_wind_ for the headwind with V_wind_ = 10 km/h. However, T_wind_ is only 2 Nm based on the calculated parameters in Table 2. The influence of the windage T_wind_ is relatively less than the friction torque analyzed in Figure 4.

In the case of a tailwind with V_wind_ = −10 km/h, the airflow can be used to generate an assisted torque. However, as shown in (5), once V_bike_ exceeds V_wind_, the assisted torque is converted to resistive torque. Nevertheless, the T_wind_ is sufficiently low during tailwind conditions.

### 3.4. Climbing-Reflected Torque

It is noted that an additional torque load is present in E-bikes during trekking conditions. As seen from the force diagram in Figure 3, the weight of the E-bike and cyclist lead to the climbing-reflected torque T_slope_ once the slope angle θ_slope_ ≠ 0. Depending on the slope angle, the T_slope_ can be shown to be:

(7) $$T_{slope}=\mathrm{mg}\times {\sin\theta }_{\mathrm{slope}}\times R_{w}$$

**Figure 6:**
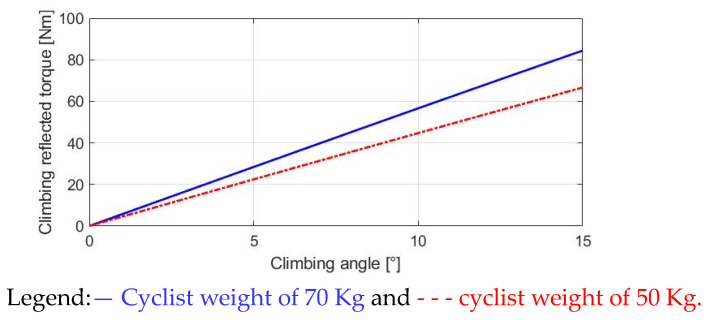
Analysis of climbing-reflected torque.

Figure 6 investigates different values of the T_slope_ with respect to the slope angle. Different from T_roll_ in (4) and T_wind_ in (6), the climbing torque T_slope_ is only dependent on the slope angle and cyclist weight. Comparing two different cyclists of 70 kg and 50 kg on the same bike, the heavier cyclist results in a higher T_slope_. However, compared to the T_roll_ simulation in Figure 4, T_slope_ is mainly affected by the slope angle θ_slope_ instead of the cyclist’s weight. As a result, the motor-assisted torque should be manipulated with respect to the slope angle θ_slope_ for different E-bike trekking conditions.

### 3.5. E-Bike Dynamic Model

After obtaining three external torque loads analyzed in Figure 3, the actual wheel driving torque T_drv_, the wheel angular acceleration α_w_, and the speed ω_w_ of the E-bike can be respectively modeled by (8) and (9):

(8) $$T_{drv}={\;T}_{total}-T_{dis}$$

(9) $$\alpha_{w}=\frac{T_{\mathrm{drv}}}{J_{w}}\;\mathrm{and}\;\omega_{w}=\sum_{t=0}^{t}\alpha_{w}{(t)\mathrm{dt}+\omega }_{w}\left(0\right)$$

where J_w_ is the corresponding wheel inertia. Considering the E-bike with different cyclist weights, J_w_ can be modeled by:

(10) $$J_{w}=\frac{1}{2}\times {(M}_{e}{+M}_{c})\times R_{w}{}^{2}$$

In (10), M_e_ and M_c_ are, respectively, the weight of the E-bike and the cyclist.

**Figure 7:**
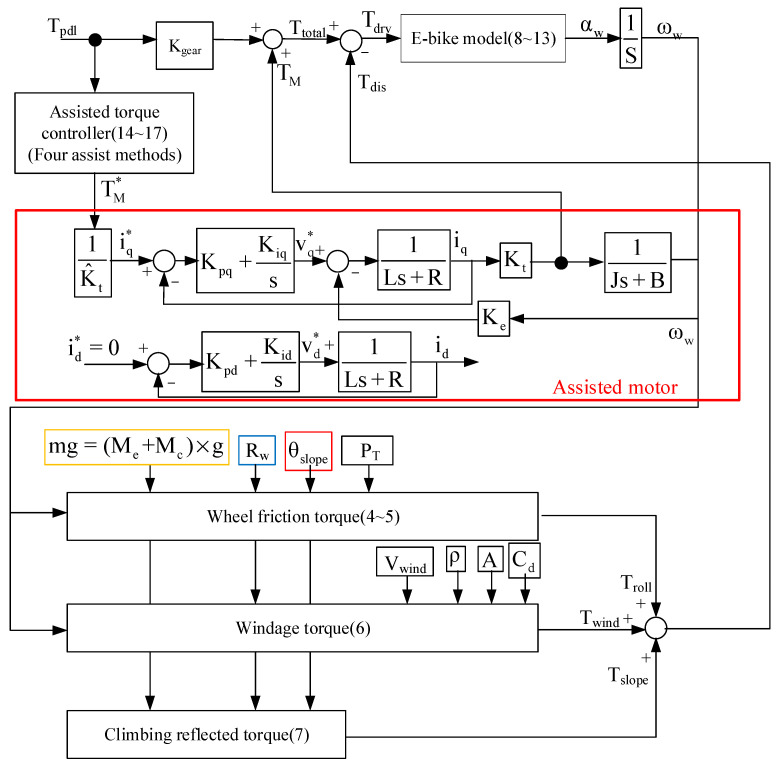
Block diagram of E-bike model control process.

For the analyzed E-bike system, the external torque loads (4)~(7) are all modeled as torque disturbances T_dis_. It is noted that the torque control for this E-bike system is equivalent to an open-loop control system in this paper. As seen in Figure 7, the total torque input T_total_ consists of the cyclist pedaling torque T_pdl_ and the motor torque T_M_. In this paper, the motor torque magnitude is manually adjusted. When the external load is increased, the cyclist is expected to generate more pedaling torque as well. In this case, the overall control stability of the assisted E-bike system is only dependent on the motor torque regulation.

Figure 7 also illustrates the corresponding torque regulation. Based on the electromagnetic energy conversion, the motor torque can be modeled by (11) in the S-domain:

(11) $$\frac{T_{M}(s)}{T_{M}{}^{*}(s)}=\frac{i_{q}(s)}{i_{q}{}^{*}(s)}=\frac{\frac{K_{pq}s+K_{iq}}{s}\frac{1}{Ls+R}}{1+\frac{K_{pq}s+K_{iq}}{s}\frac{1}{Ls+R}}$$

where i_d_, i_q_ are the stator current of the d- and q-axis. V_d_, V_q_ are the stator voltage of the d- and q-axis, K_pd_ and K_pq_ are the corresponding proportional gains, and K_id_ and K_iq_ are the corresponding integral gains.

The motor torque control is achieved based on the current field-oriented control [43]. The d-axis current i_d_ is controlled to be zero, and the torque T_M_ is directly proportional to the q-axis current i_q_. Regarding the torque controller design, pole/zero cancellation technology is used. PI controller gains are designed to be equal to:

(12) $$K_{pd}=K_{pq}=\hat{L}K_{id}=K_{iq}=\hat{R}$$

where $\hat{L}$ and $\hat{R}$ are, respectively, the estimated motor inductance and resistance parameters. Assuming ideal parameter estimation, the resulting transfer function can be modified by:

(13) $${\frac{T_{M}\left(s\right)}{T_{M}{}^{*}\left(s\right)}=\frac{W_{b}}{{s+W}_{b}}|}_{\begin{array}{l} K_{p}=\hat{L}\\ K_{i}=\hat{R} \end{array}}$$

Based on this controller design, the motor torque control can be stably maintained under external loads. In the future, a feedback-based, motor-assisted torque regulation similar to the unmanned helicopter in [44] will be investigated. Since the external friction and windage torque load are time-variant, the feedback linearization approach can be a potential solution.

## 4. Proposed E-Bike Torque Control

This section shows the simulation results for different torque control methods considering prior external loads including the wheel friction torque, windage torque, and climbing-reflected torque. Three key cycling performance indices are used to evaluate different motor torque controllers. These indices are the total torque output T_total_, wheel acceleration α_w,_ and speed ω_w_.

Key simulation parameters are listed in Table 2. The E-bike transmission gear ratio is 44 to 14 teeth, resulting in a gear ratio of 3:14. In the following simulation, MATLAB/Simulink was used to establish a simulation model in which the ideal cyclist pedaling torque T_pdl_ in Figure 1b is used. Figure 7 illustrates the control process of the E-bike model. Four motor torque-assisted methods are implemented. These four assisted methods are individually added to the original pedaling torque under the E-bike model in (3). After obtaining the total synergetic torque T_total_, the actual torque can be obtained under the influence of three external load torques. The actual wheel driving torque T_drv_, angular acceleration α_w_, and speed ω_w_ are obtained from Equations (8)–(10). It is noted that the E-bike cycling performance can be evaluated based on the E-bike wheel speed ω_w_ and acceleration α_w_ conditions.

### 4.1. No Motor-Assisted Torque (NMT)

**Figure 8:**
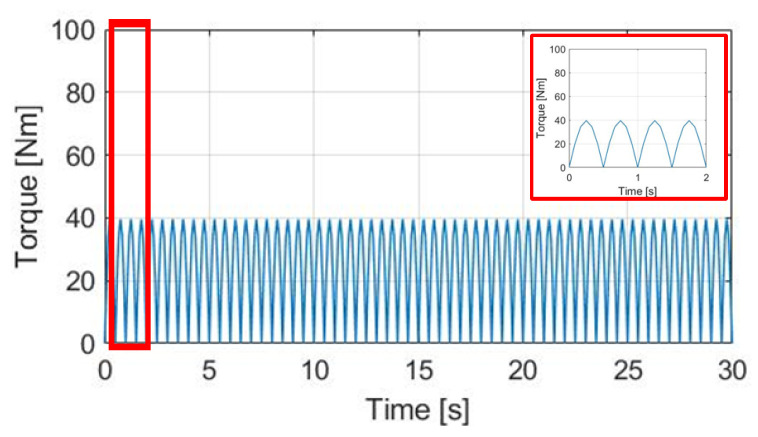
Simulation of pedaling torque versus time under 30 cpm cadence.

Normal E-bike cycling without the motor-assisted torque is first analyzed. Figure 8 shows the corresponding pedaling torque based on the torque equation in (1). In this simulation, the average pedaling torque is set at 30 Nm, with a cadence per minute of 30 cpm. The average pedaling torque transmitted to the wheel is 24.60 Nm.

**Figure 9:**
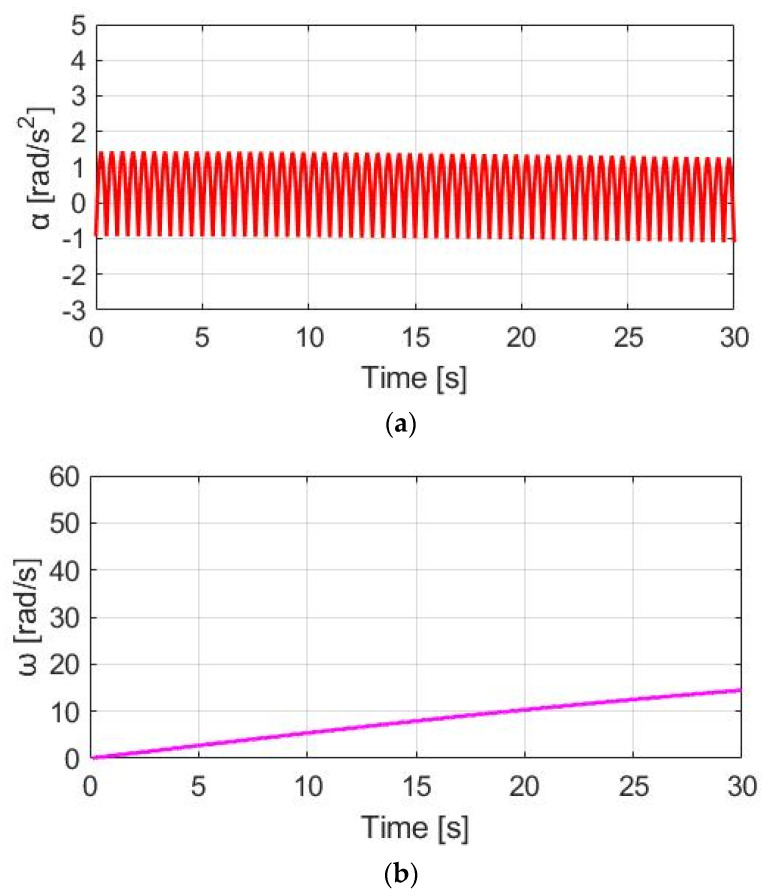
Bike dynamics based on NMT: (**a**) wheel angular acceleration; (**b**) wheel angular speed.

The simulation conditions include no wind, with a 0% slope and 70 kg cyclist weight. For the wheel acceleration α_w_ simulation in Figure 9a, the α_w_ wheel acceleration waveform is the same as the pedaling torque T_pdl_, since α_w_ is directly proportional to T_pdl_. Considering the wheel inertia J_w_ = 5.80 kg/$m^{2}$ with M_c_ = 70 kg, the average α_w_ is 0.48 rad/s^2^ with a peak-to-peak acceleration ripple of 2.38 rad/s^2^. By contrast, a wheel speed ω_w_ simulation based on (8) is also analyzed in Figure 9b. The average speed is 7.69 rad/s, with a 0.28 rad/s peak-to-peak speed ripple. The corresponding α_w_ and ω_w_ waveforms in Figure 9 can be used as a benchmark to compare the different torque control methods listed below.

### 4.2. Constant Motor-Assisted Torque (CT)

**Figure 10:**
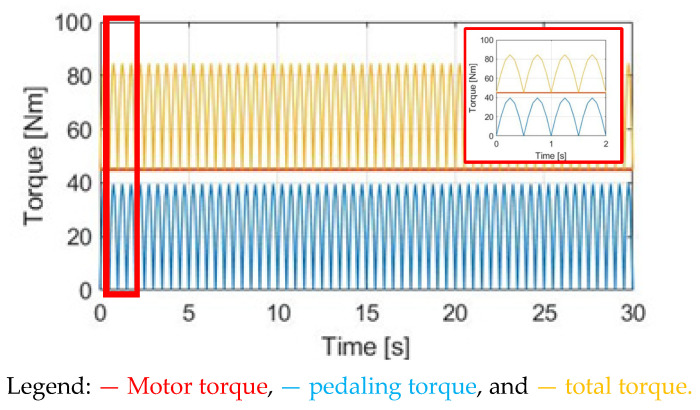
Torque comparison under the CT method.

In this section, a torque control method with a constant motor torque (CT) is applied. Figure 10 compares T_M_, T_pdl_, and T_total_ under the same 30 cpm cadence. The motor-rated torque is 45 Nm. Considering the average pedaling torque after the transmission, the ratio between T_M_ and T_pdl_ is T_M_ = 1.83 T_pdl._ To easily compare different torque waveforms, a zoom-in figure is also added in Figure 10 in this simulation.

**Figure 11:**
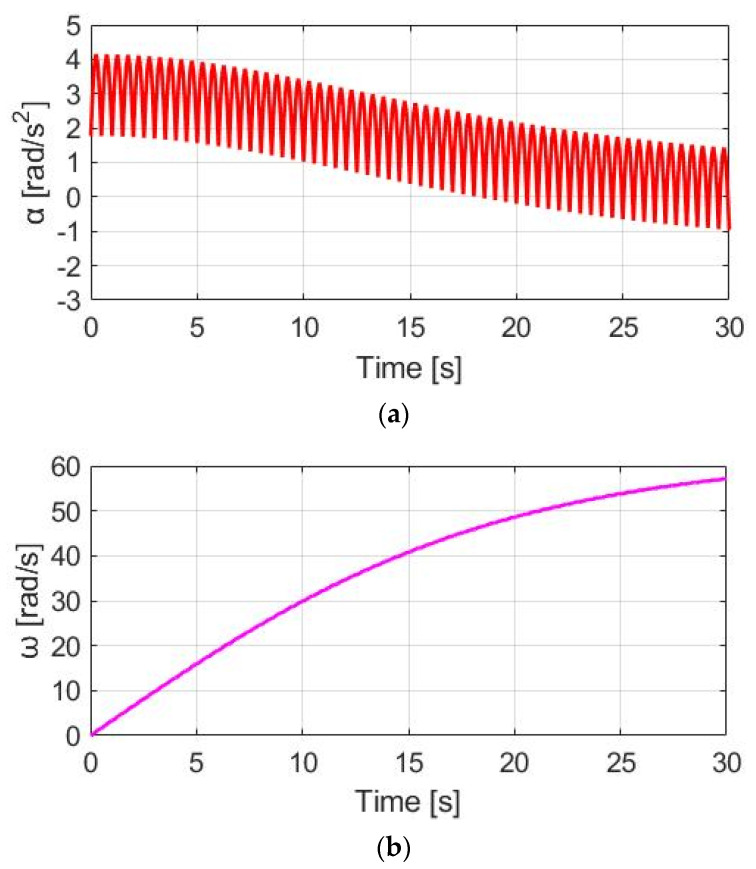
Bike dynamics based on CT: (**a**) wheel angular acceleration; (**b**) wheel angular speed.

Figure 11a shows the α_w_ for E-bike torque control with the CT method. Due to the additional constant T_M_, the average α_w_ is increased from 0.48 to 1.90 rad/s^2^. For the speed simulation in Figure 11b, the average ω_w_ is increased due to the additional T_total_. It is noteworthy that the ω_w_ ripple is increased to 0.60 rad/s compared to NMT due to the higher average α_w_ based on (8). By applying the CT method, it is concluded that both the average α_w_ and ω_w_ can be increased for a better E-bike trekking performance. However, the visible ripple in ω_w_ might degrade the cyclist’s riding experience.

### 4.3. Same Phase as Pedaling Torque (SPPT)

Instead of the CT method, this section proposes a dynamic torque control method. Under these conditions, the motor torque is manipulated by the same phase as the pedaling torque (SPPT). Based on this proposed SPPT control method, the motor torque T_M_SPPT_ is manipulated by:

(14) $$T_{M\_SPPT}=T_{M\_rated}\times \left(\frac{T_{pdl}}{T_{pdl\_peak}}\right)$$

where T_pdl_ and T_pdl_peak_ are, respectively, the instantaneous and peak value of the pedaling torque, depending on the pedaling torque sensor performance. Further, T_M_rated_ is the rated motor torque.

**Figure 12:**
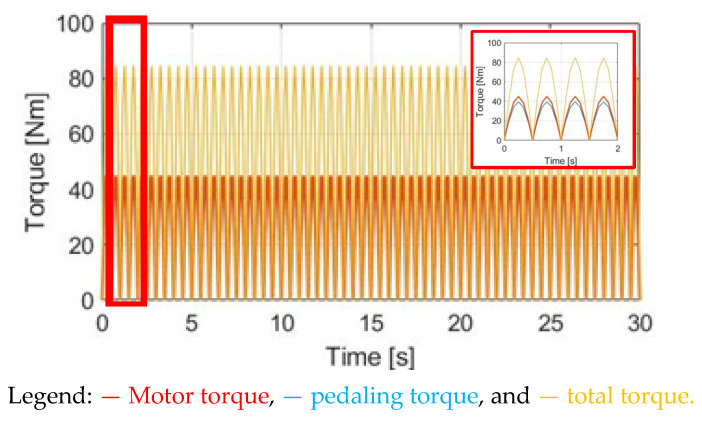
Torque comparison under the SPPT method.

**Figure 13:**
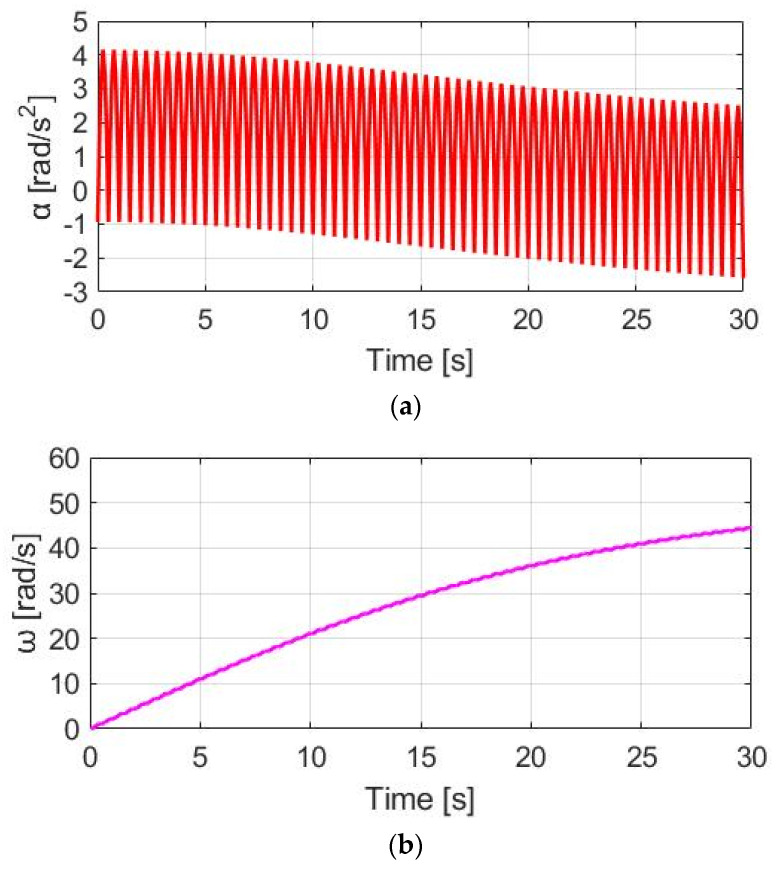
Bike dynamics based on SPPT: (**a**) wheel angular acceleration; (**b**) wheel angular speed.

Figure 12 demonstrates T_M_SPPT_, T_pdl_, and T_total_ under the same 30 cpm cadence. Comparing T_M_SPPT_ with the CT in Figure 10, it is seen that the average T_M_SPPT_ can be smaller, leading to better battery usage. However, Figure 13a demonstrates the corresponding α_w_ resulting from the SPPT method. Compared to α_w_ based on the CT method in Figure 11a, the average α_w_ is reduced from 1.90 to 1.48 rad/s^2^, but with the ripple increased from 2.41 to 5.09 rad/s^2^. For the ω_w_ speed waveform in Figure 13b, a similar decline in performance is also observed. A detailed performance comparison between the CT and SPPT methods will be explained in Section 4.6.

### 4.4. Delay Phase as Pedaling Torque (DPPT)

This section proposes another dynamic torque control method. On this basis, the ripple on the total torque can be reduced by manipulating the motor torque with a 90° delay phase as the pedaling torque (DPPT). Under this effect, the DPPT motor torque T_M_DPPT_ is formulated by:

(15) $$T_{M\_DPPT}=T_{M\_rated}\times \left(\frac{T_{pdl\_d}}{T_{pdl\_peak}}\right)$$

where T_pdl_d_ is a 90° delay torque with respect to the measured instantaneous T_pdl_. For real-time implementation, T_pdl_d_ can be obtained by:

(16) $$T_{pdl\_d}\left(90\leq \theta_{\mathrm{crank}}\leq 180\right)=T_{pdl}\left(0\leq \theta_{\mathrm{crank}}\leq 90\right)\;T_{pdl\_d}\left(180\leq \theta_{\mathrm{crank}}\leq 270\right)=T_{pdl}\left(90\leq \theta_{\mathrm{crank}}\leq 180\right)\;T_{pdl\_d}\left(270\leq \theta_{\mathrm{crank}}\leq 360\right)=T_{pdl}\left(90\leq \theta_{\mathrm{crank}}\leq 180\right)\;T_{pdl\_d}\left(0\leq \theta_{\mathrm{crank}}\leq 90\right)=T_{pdl}\left(270\leq \theta_{\mathrm{crank}}\leq 360\right)$$

It is noted that T_pdl_d_ can only be obtained after a 90° delay of θ_crank._ Due to this limitation, the E-bike might not be able to provide the motor-assisted torque during the initial startup. Nevertheless, the motor torque control can be operated after one-fourth of the pedaling cycle.

**Figure 14:**
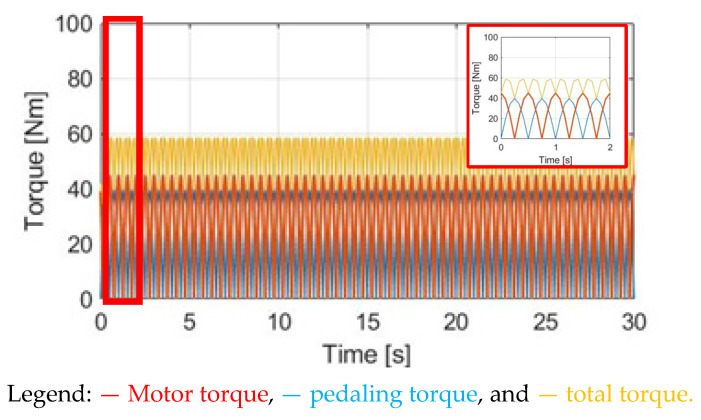
Torque comparison under the DPPT method.

**Figure 15:**
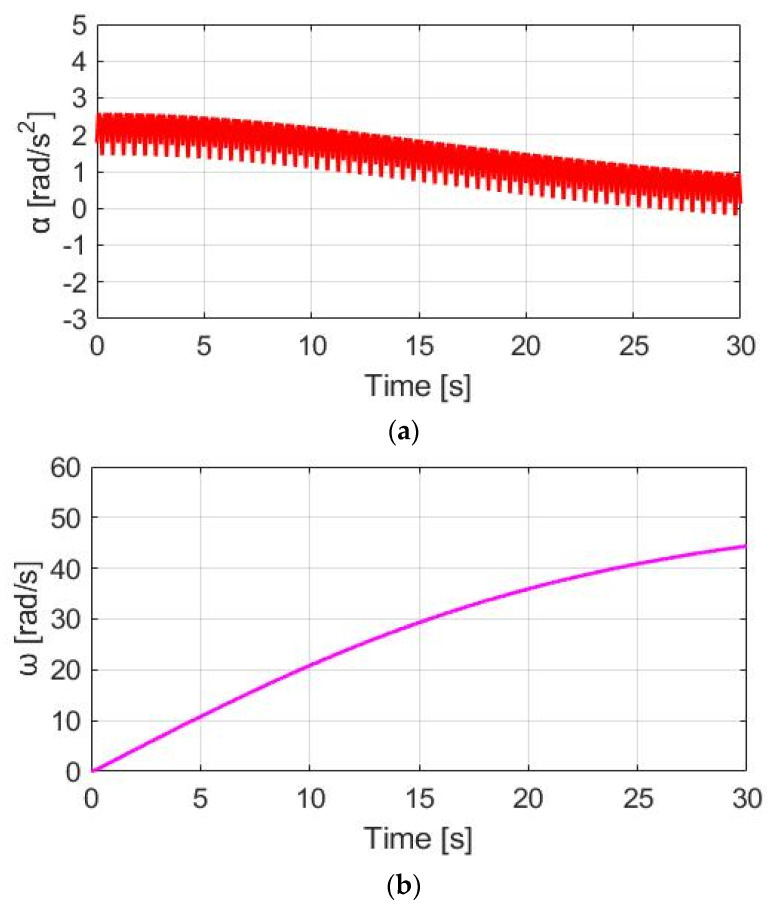
Bike dynamics based on DPPT: (**a**) wheel angular acceleration; (**b**) wheel angular speed.

Figure 14 compares T_M_DPPT_, T_pdl_, and T_total_ under the same cadence and slope situation. Since the motor torque magnitude is the same as the SPPT method, the average total torque should be the same. More importantly, because of the lower torque ripple for T_total_ in Figure 14, peak-to-peak ripples are decreased for α_w_ in Figure 15a and ω_w_ in Figure 15b. It is expected that a relatively comfortable cyclist performance is achieved. However, in Figure 15, a certain amount of T_total_ ripple is still observed, because T_pdl_ cannot be equal to the motor T_M_DPPT_. The T_total_ ripple should be increased due to the increase in T_pdl_ under the same rated motor torque T_M_rated_. A detailed comparison of the performance with the SPPT method will also be explained in Section 4.6.

### 4.5. Compensation for the Gap in the Pedaling Torque (CGPT)

**Figure 16:**
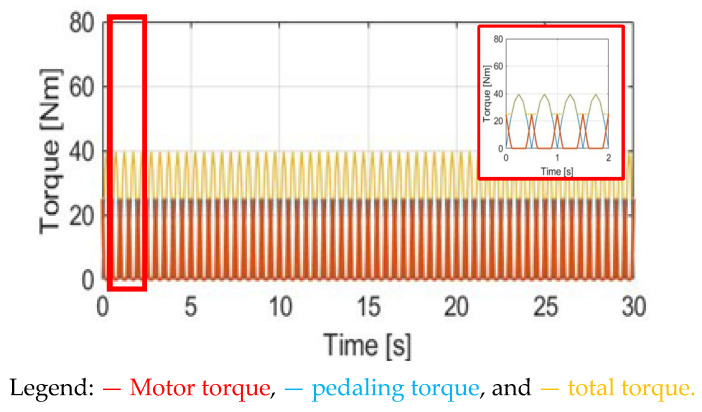
Torque comparison under the CGPT method.

This section proposes a feedback-based dynamic torque control to improve the torque ripple on the prior DPPT method. In this case, the motor torque aims to compensate for the gap in the pedaling torque (CGPT). The corresponding CGPT motor torque T_M_CGPT_ is derived from:

(17) $$\begin{array}{l} T_{M\_CGPT}=T_{M\_rated}\times \left(\frac{T_{ref}-T_{pdl}}{T_{pdl\_peak}}\right)\\ if\;T_{M\_CGPT}<0,T_{M\_\mathrm{CGPT}}=0\\ else{\;T}_{M\_\mathrm{CGPT}}{=T}_{M\_\mathrm{CGPT}} \end{array}$$

where T_ref_ is a synergy torque reference. It can be determined by the previously mentioned external load conditions. Based on the definition in (13), the manipulated motor torque T_M_CGPT_ is disabled when T_pdl_ is higher than T_ref._ By contrast, T_total_ can be the same as T_ref_ once T_pdl_ < T_ref_. Figure 16 shows the torque waveform using this CGPT method. Compared to the prior torque control methods, the primary advantage is the lowest torque ripple.

**Figure 17:**
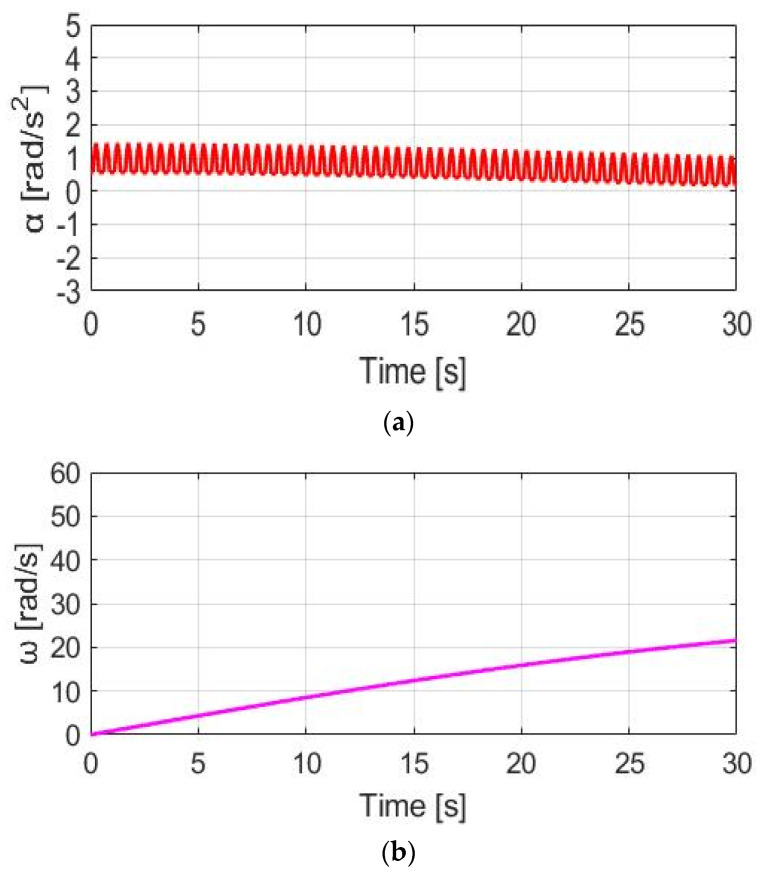
Bike dynamics based on CGPT: (**a**) wheel angular acceleration; (**b**) wheel angular speed.

Figure 17a shows the corresponding acceleration based on the CGPT method under the same simulation conditions. The α_w_ ripple is only 0.84 rad/s^2^, which is also smaller than 1.15 rad/s^2^, resulting from the prior DPPT control method. A smaller ω_w_ ripple performance can be observed in Figure 17b. However, since T_M_CGPT_ is generated only at a low T_pdl_, a drawback is the reduced average speed in Figure 17b. Comparing CT control with the highest average ω_w_ for trekking, CGPT control is well suited for commuter applications to maximize the E-bike’s battery usage.

### 4.6. Performance Comparison

Table 3 compares different torque control methods with the same cycling time. These include NMT, CT, SPPT, DPPT, and CGPT. The total synergetic torque, angular acceleration, and speed with corresponding ripples are all compared. In this comparison, the cycling time is the same as 30 s, leading to the difference in the α_w_ and ω_w_ response. By contrast, Table 4 compares these torque control methods to reach the same final speed. In Table 4, the cycling time can be different depending on different torque methods. The key findings can be summarized as follows:

(1) *CT*: The CT control method results in the highest α_w_ and ω_w_ due to the highest motor torque output. However, the ripples in α_w_ are also the highest. This method is well suited for trekking applications under visible external loads.
(2) *SPPT and DPPT:* The highest α_w_ ripple is the result of the SPPT method. When the α_w_ ripple is much higher than in the NMT case, the cyclist may have an uncomfortable experience. By contrast, for the DPPT method, a smaller α_w_ ripple is achieved under the same motor torque. Compared to SPPT control, the DPPT method can provide a comparable cycling experience as the original NMT. The DPPT method is well suited for standard E-bike torque management for different load conditions.
(3) *CGPT:* Because the CGPT method generates the lowest motor torque, the resulting α_w_ ripple can be smaller than the original NMT condition. However, the lowest motor output might degrade the E-bike’s acceleration performance. As seen in Table 4, CGPT requires 18.72 s to reach a 15 rad/s final speed. By contrast, for CT control, only 4.72 s is spent. It is concluded that the CGPT is well suited for commuting cyclists. This control results in the best battery usage at the smallest α_w_ ripple. It is especially well suited for cyclists under a heavy daily urban traffic burden.

## 5. Experiment

**Figure 18:**
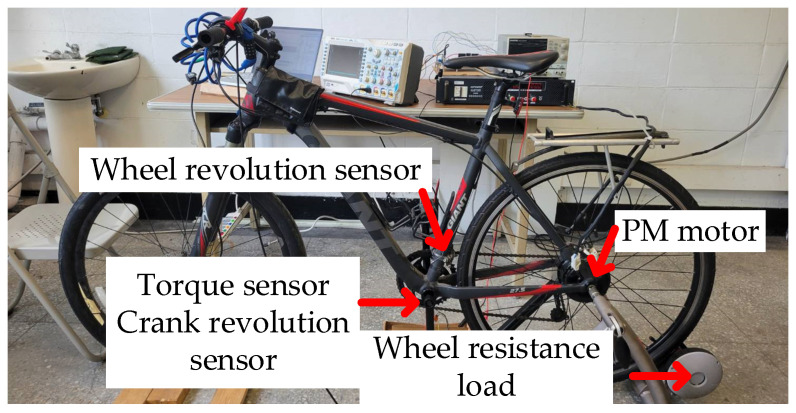
E-bike experimental test setup and sensor hardware installation.

This section describes the experimental verification. Figure 18 shows a photograph of the E-bike experimental test setup. The experiment is performed based on a 250 W 300 rpm permanent magnet (PM) AC motor. Field-oriented control (FOC) through the Hall sensor position feedback is implemented. As seen in Figure 18, the PM motor is attached to the rear wheel of the E-bike. Detailed PM motor specifications are listed in Table 5. It should be noted that the experimental test setup for the E-bike is currently under laboratory verification. At this time, power supply hardware is used for the E-bike’s power source to provide a reliable DC voltage. The E-bike analyzed is based on a standard assisted E-bike with 250 W electrical power. Considering the actual E-bike product in the future, a Li-ion battery with 7 Amp hours can be selected to provide a comparable DC voltage.

**Figure 19:**
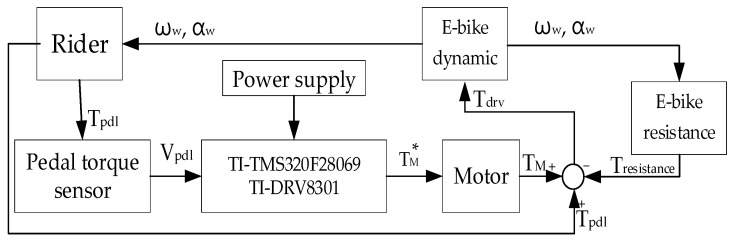
Hardware setup and signal process for E-bike torque control experiment.

**Figure 20:**
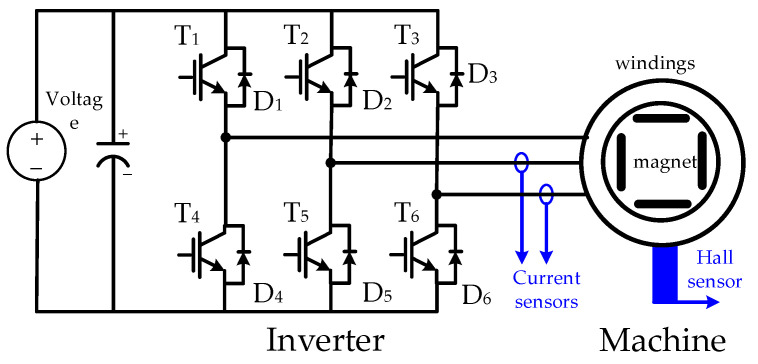
Electrical circuit of six-switch motor drive inverter.

**Figure 21:**
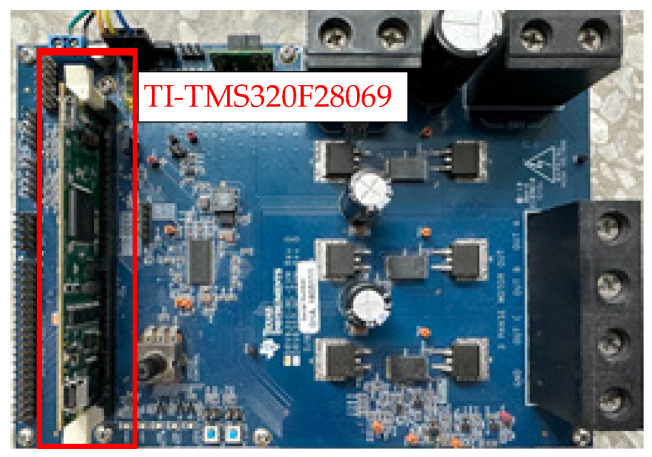
Photograph of motor drive inverter.

Figure 19 illustrates the hardware setup and signal process for the E-bike torque control experiment. All four different motor torque control methods are implemented on a 32-bit microcontroller, TI-TMS320F28069. The interrupt service routine is designed at 10 kHz, which is synchronous with the sampling frequency. In addition, the motor drive inverter is selected with the TI-DRV8301 evaluation kit. On this basis, the E-bike motor can be controlled through a six-switch pulse-width modulation inverter, as shown in Figure 20. Figure 21 illustrates a photograph of the test motor drive inverter, the TI-DRV8301 evaluation kit. This inverter kit can be easily integrated with the TI-TMS320F28069 microcontroller used as a motor drive system. In this case, the motor-assisted torque can be manipulated based on the desired torque command mentioned in Figure 7.

For the PM motor control, three-phase pulse width modulation voltages are manipulated by the controller for the motor to generate the desired torque output. Because the FOC requires the instantaneous position for sinusoidal voltage control, zero-order hold (ZOH) position interpolation [45,46,47,48] is used to improve the Hall-based position-sensing resolution. As seen in (18), the position interpolation is performed every 60°:

(18) $$\begin{array}{l} {\hat{\theta }}_{k}(t)=\theta_{k-1}+{\hat{\omega }}_{k-1}(t_{k}-t_{k-1})\\ \theta_{k-1}\leq {\hat{\theta }}_{k}(t)\leq \theta_{k-1}+\frac{\pi }{3} \end{array}$$

where ${\hat{\theta }}_{k}$ and $\theta_{k-1}$ are, respectively, the estimated current motor position and the last position measured by Hall sensors. Further, ${\hat{\omega }}_{k-1}$ is the estimated speed based on prior Hall sensor position information, and $t_{k}$ and $t_{k-1}$ are, respectively, the current and last time interval. The estimated speed ${\hat{\omega }}_{k-1}$ can be obtained by:

(19) $${\hat{\omega }}_{k-1}=\frac{\pi /3}{\Delta t_{k-1}}=\frac{\pi /3}{t_{k-1}-t_{k-2}}$$

In (19), ${\hat{\omega }}_{k-1}$ is calculated based on the two prior time steps, $t_{k-1}$ and $t_{k-2}$. It is noted that the position interpolation is under the average speed assumption in (19) without instantaneous motor acceleration and deceleration. For E-bike applications, this assumption is still valid during normal cycling conditions.

Regarding the pedaling torque and crank cadence measurement, both the torque sensor and crank position sensor are installed inside the bracket bottom. Considering E-bike operation under different external loads, a wheel-resistive load in Figure 18 is added on the rear wheel for the load simulation. In the experiment, the pedaling torque sensor can transmit a voltage reference of between 0.7–3.3 v to the microcontroller. This voltage reference is proportional to a pedaling torque of 0–80 Nm. The experimental verification compares the cycling performance among normal NMT and the four torque control methods. However, for actual riding conditions, it is not possible for a cyclist to maintain the same pedaling torque under different load and assisted torque conditions. Under this effect, the test cyclist in these experiments was asked to maintain a wheel speed ω_w_ of 15.88 rad/s (20 km/h). If ω_w_ can be maintained at a more stable speed without variation, the motor torque is assumed to assist the cyclist.

### 5.1. NMT and CT Experiment

**Figure 22:**
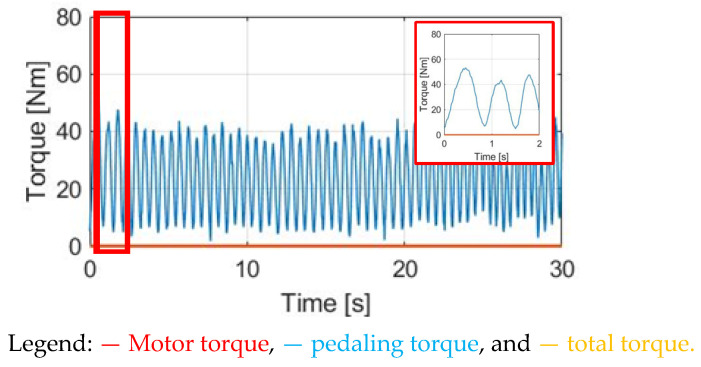
Torque comparison under the NMT.

**Figure 23:**
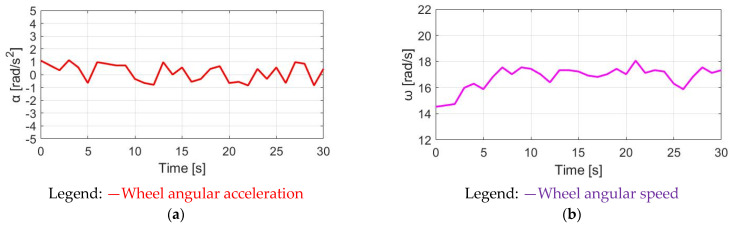
NMT-reflected E-bike response: (**a**) wheel angular acceleration and (**b**) wheel angular speed.

This section compares the time-domain waveforms of the pedaling torque, motor torque, and total synergy torque between normal NMT and the CT motor control. Since there is no assisted torque under the NMT method, the test cyclist was responsible for different E-bike load conditions. Figure 22 and Figure 23, respectively, show the pedaling torque, α_w_, and ω_w_ waveforms under normal NMT. In this case, the corresponding pedaling torque condition can be used as a benchmark to compare the four different torque control methods.

In this experiment, a wheel resistive load was added to simulate E-bike cycling with wheel friction torque. Under a certain wheel friction load, the pedaling torque measured from the torque sensor contains a 29.81 Nm average torque with 59.92% pedaling torque variation. For all experiments in this paper, the pedaling torque variation $T_{pdl\_r}$ is defined by:

(20) $$T_{pdl\_r}=\frac{T_{pdl\_max}-T_{pdl\_avg}}{T_{pdl\_avg}}$$

where $T_{pdl\_max}$ and $T_{pdl\_avg}$ are, respectively, the maximum and average value of the measured pedaling torque $T_{pdl}$.

**Figure 24:**
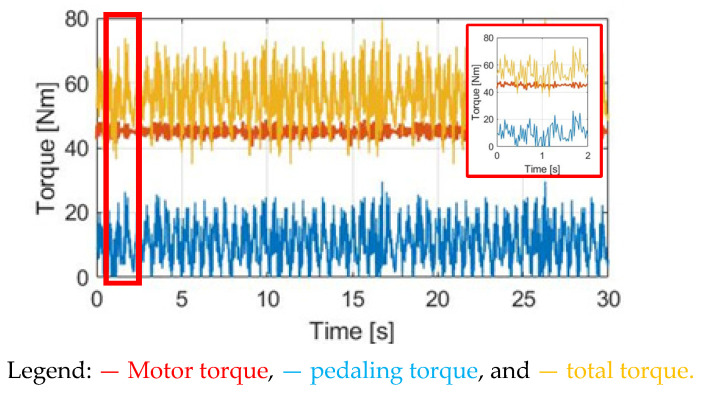
Torque comparison under CT control.

**Figure 25:**
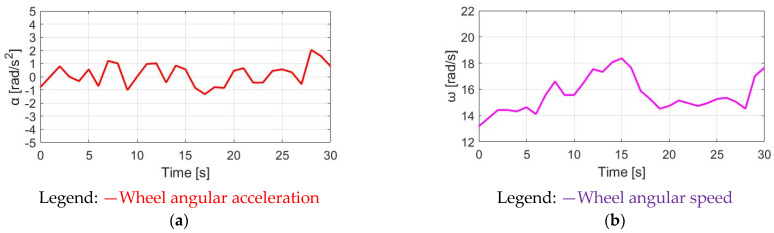
CT-reflected E-bike response: (**a**) wheel angular acceleration and (**b**) wheel angular speed.

By contrast, considering the CT control method, Figure 24 demonstrates the time-domain waveforms of $T_{pdl}$, the motor torque $T_{M},$ and the total torque $T_{total}$. Figure 25 shows the time-domain waveforms of α_w_ and ω_w_. In this control, the motor torque is controlled to maintain a 45 Nm rated torque. With additional assisted torque, the resulting average pedaling $T_{pdl}$ is reduced to 13.05 Nm. However, similar to the prior simulation, the pedaling variation $T_{pdl\_r}$ is increased to 63.86% due to the limitation on the constant motor torque regulation. The key differences in the performance of the torque are summarized in Section 5.4.

### 5.2. SPPT and DPPT Experiment

**Figure 26:**
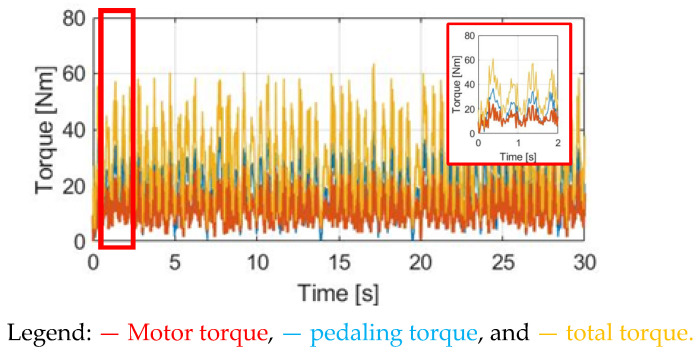
Torque comparison under SPPT control method.

**Figure 27:**
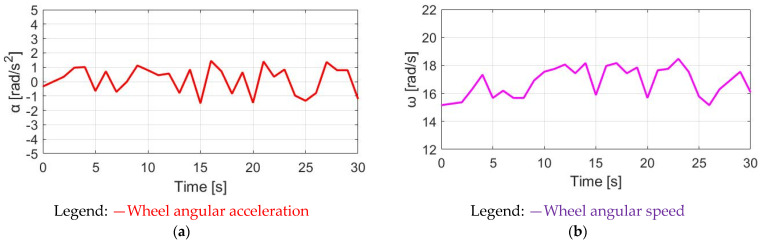
SPPT-reflected E-bike response: (**a**) wheel angular acceleration and (**b**) wheel angular speed.

**Figure 28:**
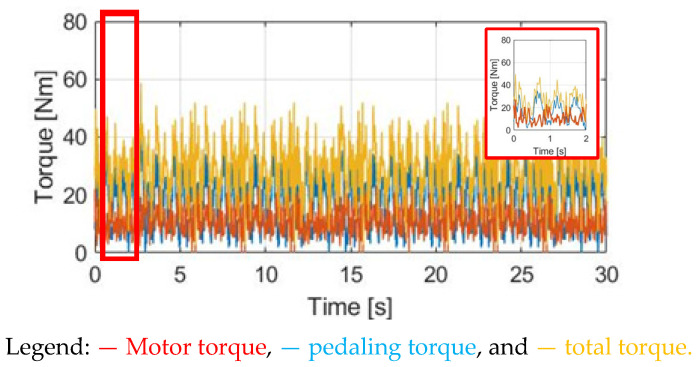
Torque comparison under DPPT control method.

**Figure 29:**
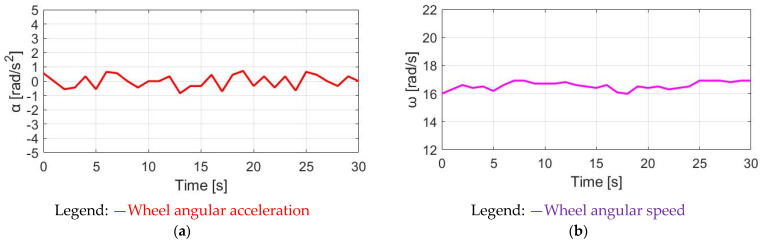
DPPT-reflected E-bike response: (**a**) wheel angular acceleration and (**b**) wheel angular speed.

Figure 26 and Figure 27 depict the measurements of torque and bike dynamics, respectively, obtained under the SPPT condition. Similarly, Figure 28 and Figure 29 illustrate the corresponding measurements of torque and bike dynamics collected under the DPPT condition.

In addition, Figure 26 and Figure 28 compare the waveforms of the pedaling $T_{pdl}$, motor $T_{M},$ and total $T_{total}$ under the dynamic SPPT and DPPT control methods, respectively. Since the motor torque $T_{M}$ is dynamically controlled under the SPPT and DPPT methods, $T_{M}$ is calculated proportionally to the measured pedaling torque $T_{pdl}$ for the SPPT method in (14) and the DPPT method in (15).

It is noted that for the DPPT method, the time-domain waveform of the motor torque $T_{M}$ is delayed by 90° with respect to the measured pedaling $T_{pdl}$. Considering the same E-bike external load, the cyclist that is reflected by pedaling $T_{pdl}$ is almost the same. However, under the same average assisted torque $T_{M}$, the total torque variation through DPPT is smaller than the variation reflected by SPPT, as listed in Table 6. Similar to the simulation comparison, it is expected that the variation in both α_w_ and ω_w_ are smaller, as shown in Figure 27 and Figure 29. A detailed comparison of α_w_ and ω_w_ will be explained in Section 5.5.

### 5.3. Proposed CGPT Experiment

**Figure 30:**
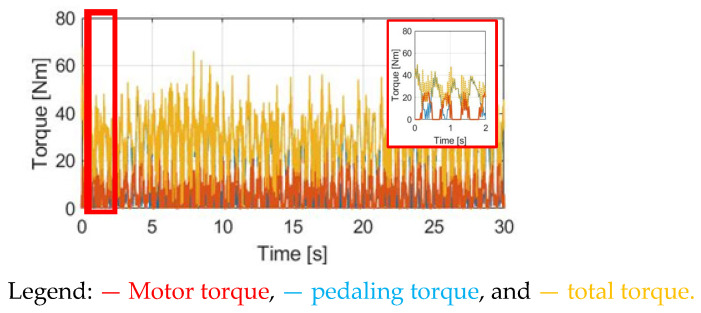
Torque comparison under CGPT control method.

**Figure 31:**
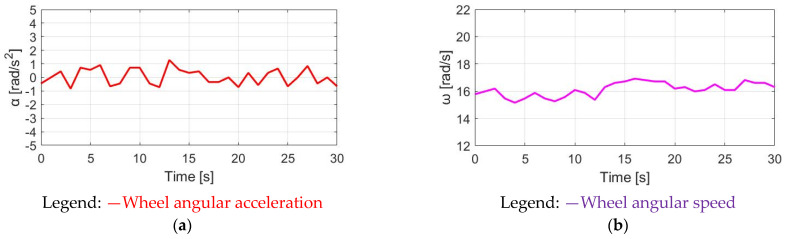
CGPT-reflected E-bike response: (**a**) wheel angular acceleration and (**b**) wheel angular speed.

Time-domain torque waveforms through the proposed CGPT control are shown in Figure 30. In addition, the time-domain α_w_ and ω_w_ waveforms are included in Figure 31. As seen in Section 4.5, the CGPT-assisted torque is determined based on (17). For the actual experiment, $T_{ref}$ is determined at 25 Nm, which is the average pedaling torque $T_{pdl}$ on the rear wheel under normal NMT. When the pedaling torque transmission to the rear wheel is smaller than 25 Nm, $T_{M}$ should be enabled similarly to the DPPT control condition. Based on the simulation, it is expected that the average and maximum motor $T_{M}$ are the lowest among the four torque control methods. This leads to better E-bike battery usage.

### 5.4. E-Bike Torque Performance Comparison

Table 6 summarizes the waveform conditions among the pedaling, motor, and total torque. For normal NMT, the average pedaling torque is 29.81 Nm, with 59.92% torque variation. By adding one of the four torque controls, the cyclist pedaling torque can be effectively decreased for better riding performance.

**Table 6:** Motor torque comparison under different torque control methods.

	Assisted Method	NMT	CT	SPPT	DPPT	CGPT
Parameter						
Average pedaling torque (Nm)		29.81	13.05	20.83	21.05	25.63
Average motor torque (Nm)		NA	45	12.01	11.93	8.24
Max pedaling torque (Nm)		74.37	36.11	48.09	48.13	62.67
Max motor torque (Nm)		NA	45	27.05	27.07	25
Pedaling torque variation (Nm)		44.56	23.06	27.26	27.08	37.04
Variation ratio (Nm/%)		59.92%	63.86%	56.69%	56.26%	59.10%
Average total torque (Nm)		29.81	58.05	32.84	32.98	33.87
Max total torque (Nm)		74.37	81.11	75.14	75.20	87.67

For the CT-assisted control method, the minimal average pedaling torque of 13.05 Nm is the result of the cyclist maintaining the wheel speed ω_w_ at 15.88 rad/s (20 km/h).

The difference between SPPT and DPPT is the torque waveform’s initial phase. Under this effect, there is no visible difference in the cyclist’s reflected pedaling torque. However, the variation in α_w_ and ω_w_ might be different due to different peak total torques with these two control methods.

By contrast, for the proposed CGPT control method, the motor torque is efficiently manipulated. However, the required pedaling torque is the highest among these four assisted control methods. This is because, similar to the DPPT method, a smooth condition for the α_w_ and ω_w_ of the E-bike is expected.

### 5.5. E-Bike Speed and Acceleration Comparison

This section compares the performance of the E-bike acceleration α_w_ and speed ω_w_ in Table 7 under the different proposed torque controls. It is noted that the average value and ripple of α_w_ and ω_w_ are both dependent on the total torque $T_{total}$ in Table 6. Since the CT-assisted control results in the highest variation in $T_{total}$, the highest ripples of both α_w_ and ω_w_ are shown in Table 7. This experimental result is consistent with the simulation in Table 3.

Although the pedaling torque condition is similar with the SPPT and DPPT methods, the variation in the total synergy torque might be different. Under this effect, the ripples of α_w_ and ω_w_ for the DPPT control are smaller than those with SPPT control. Finally, for the proposed CGPT method, the corresponding α_w_ and ω_w_ ripple is slightly higher than those with the DPPT methos. However, compared to CT and SPPT controls, the CGPT method still results in a better α_w_ and ω_w_ ripple performance for E-bike torque-assisted control.

### 5.6. Simulation and Experiment Comparison

This section compares the results obtained by both the simulation and the experiment. Table 8 shows the corresponding comparison of the simulation and the experiment under different assisted torque control methods. The key findings are summarized as follows.

First, the pedaling torque performance is compared. For the E-bike simulation, an ideal pedaling torque is assumed. Under this effect, there is no difference in the average and maximum pedaling torque among these four torque control methods. By contrast, for the experiment, the pedaling torque is directly provided by a test cyclist. Because this cyclist must maintain an overall cycling time at 30 s, the average and maximum pedaling torque are both highest under NMT control, whereas they are the smallest with CT control. From the prior conclusion in Table 6, the largest motor torque is manipulated for CT control, resulting in the lowest pedaling torque for a cyclist.

For the wheel speed and acceleration ripple comparison in Table 8, both speed and acceleration ripples can degrade the E-bike’s cycling performance. Comparing the results between the simulation and the experiment, speed/acceleration ripples are highest for the CT control method. By contrast, these ripples can be reduced based on the implementation of either DPPT or CGPT control. The proposed simulation is consistent with the experimental results.

For the average speed and acceleration comparison in Table 8, there is a difference between the simulation and the experiment. For the simulation, the average speed and acceleration are directly proportional to the average torque. By contrast, for the experiment, the average speed is almost the same under the limitation of maintaining the same cycling time. However, the average acceleration is also the highest for the CT control method, with the highest average torque.

## 6. Conclusions

This paper proposes a novel torque control method for assisted E-bikes considering external load conditions. For assisted E-bikes, it is shown that the overall pedaling torque can be affected by different load conditions. These include the cyclist’s weight, wind resistance, rolling resistance, and the road slope. Among them, the external loads caused by the road gradient and wind resistance are greater than those caused by the cyclist’s weight and the rolling resistance.

**Figure 32:**
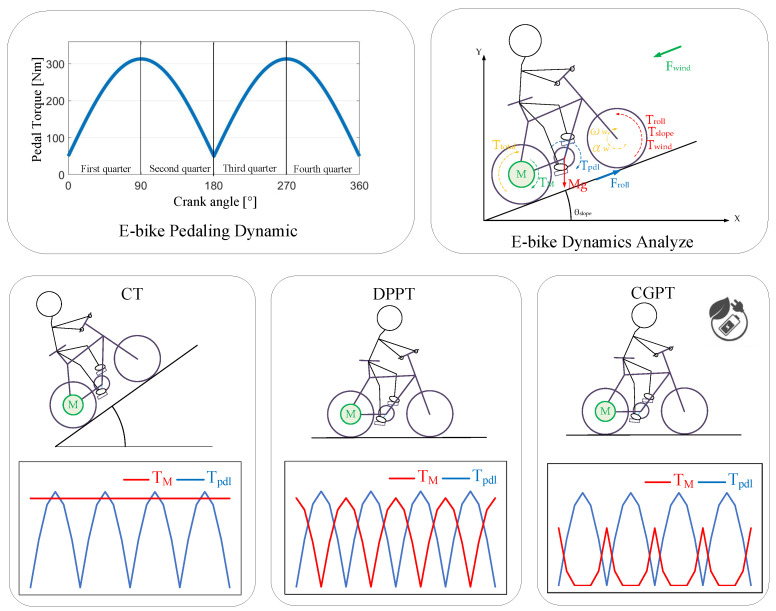
Graphical conclusion of proposed E-bike torque control.

Figure 32 illustrates a graphical conclusion of the proposed E-bike torque control. Key E-bike cycling parameters were first identified. Four different torque control methods were developed to improve the E-bike’s dynamic response with minimal pedaling torque variation and acceleration/speed ripple. After the simulation verification from MATLAB/Simulink, an integrated E-bike sensor hardware was built to evaluate the proposed torque control. Finally, the proposed assisted torque control was verified through an experimental E-bike test bench.

The experimental results conclude that the CT method achieves the smallest average pedaling torque. However, it results in the highest speed ripple and acceleration ripple. These ripples degrade the E-bike’s cycling performance. It is concluded that the CT control method is well suited for professional cyclists with special road conditions.

On the other hand, the proposed CGPT control resulted in the lowest motor torque output. It is especially well suited for commuting cyclists with minimal battery power consumption. By contrast, the DPPT control method can provide a comparable cycling experience to the original NMT method in terms of the wheel acceleration ripple and speed ripple. The DPPT method is well suited for standard E-bike torque management for different load conditions.

## Figures and Tables

**Table 1:** Key findings of existing references.

Category	References
Torque control for assisted E-bike	[1,2,24,38,39]
Instantaneous pedaling torque waveform	[4,5,6,7]
Pedaling torque component analysis	[8,9,10,11,12,13,14]
Factors affecting riding	[15,16,17,18,19,20,21,22,23,25,26,27,28,29,30,31,32,33,34,35]
Recharge control for assisted E-bike	[36,37]

**Table 2:** Assisted E-bike model parameters.

Parameter	Value
Mass of E-bike (M_e_)	25 kg
Mass of cyclist (M_c_)	70 or 50 kg
Wheel inertia (J_w_)	5.8 or 4.6 kg/$m^{2}$
Bike wheel radius (Rw)	0.35 m
Gravitational constant (g)	9.81 $m/s^{2}$
Density of air (ρ)	1.2258 kg/$m^{3}$
Aerodynamic drag coefficient ($C_{d}$) [40]	0.4
Frontal area (A)	0.645 $m^{2}$
Maximum cadence per minute	30 cpm
Transmission gear ratio ($K_{gear}$)	44/14
Tire pressure (P_T_)	32 psi

**Table 3:** Comparison of different assisted methods with the same cycling time.

	Assisted Method	NMT	CT	SPPT	DPPT	CGPT
Parameter						
Average pedaling torque (Nm)		30	30	30	30	30
Average motor torque (Nm)		N/A	45	27.91	27.91	5.37
Max motor torque (Nm)		N/A	45	45	45	25
Speed ripple (rad/s)		0.28	0.60	0.55	0.15	0.10
Average speed (rad/s)		7.69	36.44	26.89	26.89	11.82
Acceleration ripple (rad/s^2^)		2.38	2.41	5.09	1.15	0.84
Average acceleration (rad/s^2^)		0.48	1.90	1.48	1.48	0.72
Cycling time (s)		30	30	30	30	30

**Table 4:** Comparison of different assisted methods to reach the same final speed.

	Assisted Method	NMT	CT	SPPT	DPPT	CGPT
Parameter						
Average motor torque (Nm)		N/A	45	27.91	27.91	5.37
Max motor torque (Nm)		N/A	45	45	45	25
Final speed (rad/s)		15.00	15.00	15.00	15.00	15.00
Average acceleration (rad/s^2^)		0.48	3.18	2.19	2.19	0.80
Required time s)		31.32	4.72	6.86	6.86	18.72

**Table 5:** Specification of E-bike PM motor.

Parameter	Value
Rated voltage	36 V
Maximum torque	45 Nm
Rated power	250 W
Weight	2.46 kg
Outer radius	129 mm
Maximum speed	3000 rpm
Installation location	Rear wheel

**Table 7:** E-bike speed and acceleration comparison under different torque control methods.

	Assisted Method	NMT	CT	SPPT	DPPT	CGPT
Parameter						
Speed ripple (rad/s)		3.43	4.56	3.32	0.94	1.76
Average speed (rad/s)		16.84	15.56	16.85	16.59	16.14
Acceleration ripple (rad/s^2^)		1.97	3.37	2.96	1.56	2.12
Average acceleration (rad/s^2^)		0.16	1.20	0.16	0.16	0.07
Cycling time (s)		30	30	30	30	30

**Table 8:** Comparison between simulation and experiment under different torque control methods.

	Assisted Method	NMT		CT		SPPT	
Parameter		(sim.)	(exp.)	(sim.)	(exp.)	(sim.)	(exp.)
Average pedaling torque (Nm)		30	29.81	30	13.05	30	20.83
Max pedaling torque (Nm)		48.78	74.37	48.78	36.11	48.78	48.09
Average motor torque (Nm)		N/A	N/A	45	45	27.91	12.01
Max motor torque (Nm)		N/A	N/A	45	45	45	27.05
Speed ripple (rad/s)		0.28	3.43	0.60	4.56	0.55	3.32
Acceleration ripple (rad/s^2^)		2.38	1.97	2.41	3.37	5.09	2.96
Average speed (rad/s)		7.69	16.84	36.44	15.56	26.89	16.85
Average acceleration (rad/s^2^)		0.48	0.16	1.90	1.20	1.48	0.16
Cycling time (s)		30	30	30	30	30	30
	**Assisted Methods**	**DPPT**			**CGPT**		
**Parameters**		**(sim.)**	**(exp.)**		**(sim.)**	**(exp.)**	
Average pedaling torque (Nm)		30	21.05		30	25.63	
Max pedaling torque (Nm)		48.78	48.13		48.78	62.67	
Average motor torque (Nm)		27.91	11.93		5.37	8.24	
Max motor torque (Nm)		45	27.07		25	25	
Speed ripple (rad/s)		0.15	0.94		0.10	1.76	
Acceleration ripple (rad/s^2^)		1.15	1.56		0.84	2.12	
Average speed (rad/s)		26.89	16.59		11.82	16.14	
Average acceleration (rad/s^2^)		1.48	0.16		0.72	0.07	
Cycling time (s)		30	30		30	30	

## Data Availability

No new data were created or analyzed in this study. Data sharing is not applicable to this article.

## Abbreviations

θ_crank_	Crank rotating angle
F_px_	Pedaling horizontal force
F_py_	Pedaling vertical force
$T_{pdl}$	Pedaling torque
T_M_	Motor-assisted torque
T_total_	Total synergetic torque
T_roll_	Friction-reflected torque
T_wind_	Windage torque
T_slope_	Climbing-reflected torque
T_dis_	External disturbance torque
T_drv_	Actual wheel driving torque
$T_{pdl\_r}$	Pedaling torque variation
$T_{pdl\_max}$	Maximum measured pedaling torque
$T_{pdl\_avg}$	Average measured pedaling torque
$R_{crank}$	Crank rotating radius
M_e_	Mass of E-bike
M_c_	Mass of cyclist
J_w_	Wheel inertia
R_w_	Bike wheel radius
g	Gravitational constant
ρ	Air density
$C_{d}$	Aerodynamic drag coefficient
A	Frontal area
$K_{gear}$	Transmission gear ratio
P _T_	Tire pressure
*ω_w_*	Wheel angular speed
*α_w_*	Wheel angular acceleration
K_roll_	Wheel resistance coefficient
V_wind_	Wind speed
V_bike_	Bike wheel speed
θ_slope_	Slope angle
i_d_	Direct-axis (d-axis) motor current
i_q_	Quadrature-axis (q-axis) motor current
V_d_	Direct-axis (d-axis) motor voltage
V_q_	Quadrature-axis (q-axis) motor voltage
K_pd_	Direct-axis (d-axis) proportional controller gain
K_pq_	Quadrature-axis (q-axis) proportional controller gain
K_id_	Direct-axis (d-axis) integral controller gain
K_iq_	Quadrature-axis (q-axis) integral controller gain
$\hat{L}$	Motor phase inductance
$\hat{R}$	Motor phase resistance
${\hat{\theta }}_{k}$	Estimated motor position
$\theta_{k-1}$	Last position measured by Hall sensors
${\hat{\omega }}_{k-1}$	Estimated speed based on prior Hall sensor position
